# In Situ Detection of Salmonid Alphavirus 3 (SAV3) in Tissues of Atlantic Salmon in a Cohabitation Challenge Model with a Special Focus on the Immune Response to the Virus in the Pseudobranch

**DOI:** 10.3390/v15122450

**Published:** 2023-12-15

**Authors:** Haitham Tartor, Lisa-Victoria Bernhardt, Saima Nasrin Mohammad, Raoul Kuiper, Simon C. Weli

**Affiliations:** 1 Department of Fish Health, Norwegian Veterinary Institute, 1433 Ås, Norway; saima-nasrin.mohammad@vetinst.no; 2 DNV Headquarters, Høvik, 1363 Bærum, Norway; lisa.victoria.bernhardt@dnv.com; 3 Department of Fish Biosecurity, Norwegian Veterinary Institute, 1433 Ås, Norway; raoul.valentin.kuiper@vetinst.no (R.K.); simon.weli@vetinst.no (S.C.W.)

**Keywords:** Atlantic salmon, salmonid alphavirus 3 (SAV3), pseudobranch, immune response, pancreas disease (PD), in situ hybridization, RNAscope^®^

## Abstract

Salmonid alphavirus strain 3 is responsible for outbreaks of pancreas disease in salmon and rainbow trout in Norway. Although the extensive amount of research on SAV3 focused mainly on the heart and pancreas (of clinical importance), tropism and pathogenesis studies of the virus in other salmon tissues are limited. Here, we used a combination of RT-qPCR (Q_nsp1 gene) and in situ hybridization (RNAscope^®^) to demonstrate the tropism of SAV3 in situ in tissues of Atlantic salmon, employing a challenge model (by cohabitation). In addition, as previous results suggested that the pseudobranch may harbor the virus, the change in the expression of different immune genes upon SAV3 infection (RT-qPCR) was focused on the pseudobranch in this study. In situ hybridization detected SAV3 in different tissues of Atlantic salmon during the acute phase of the infection, with the heart ventricle showing the most extensive infection. Furthermore, the detection of the virus in different adipose tissues associated with the internal organs of the salmon suggests a specific affinity of SAV3 to adipocyte components. The inconsistent immune response to SAV3 in the pseudobranch after infection did not mitigate the infection in that tissue and is probably responsible for the persistent low infection at 4 weeks post-challenge. The early detection of SAV3 in the pseudobranch after infection, along with the persistent low infection over the experimental infection course, suggests a pivotal role of the pseudobranch in SAV3 pathogenesis in Atlantic salmon.

## 1. Introduction

Alphaviruses (family Togaviridae) is a diverse group of small, spherical, enveloped viruses with single-stranded, positive-sense RNA genomes [1]. 

So far, alphaviruses that infect salmonids (SAV) show six SAV subtypes (SAV1–SAV6) based on the nucleic acid sequences encoding two of the virus proteins (E2 and nsP3) [2]. The different subtypes of the virus are known to cause different diseases in salmonids and have been seen as an increasing problem in the European salmonid-farming industry [3]. SAV1, for example, is the causative agent of pancreas disease (PD) in Atlantic salmon (*Salmo salar* L.) in the British Isles [4]. SAV2 is divided into two subgroups: the freshwater (FW) variant, SAV2 FW, which causes sleeping disease in rainbow trout (*Oncorhynchus mykiss*, Walbaum) in freshwater in France [5], England [6], and several European countries [7]; and the marine variant, marine SAV2, which causes PD in seawater-reared Atlantic salmon (*Salmo salar* L.) and rainbow trout in mid and north of Norway. SAV3, on the other hand, has been detected only in Norway (North and South) and is responsible for outbreaks of PD in Atlantic salmon and rainbow trout [8]. Moreover, while SAV4 and SAV6 have been identified in association with Irish PD outbreaks, SAV5 is seen in conjunction with the disease outbreaks in Scotland. 

Salmonid alphavirus 3 tropism in Atlantic salmon has been previously studied in an experimental infection model with an atypical infection route (intraperitoneal injection [i.p.]) using RT-qPCR [3]. However, the precise in situ localization of SAV3 in tissues of cohabitant-infected salmon has not yet been studied. Histopathological studies of PD in Atlantic salmon showed lesions predominantly in the pancreas, heart, and skeletal muscle [9]. Despite the reported persistence of SAV3 in gills and pseudobranch [3], histopathological changes were not reported. Also, the immunological studies addressing SAV3 infection in salmon focused on the heart, peritoneal cavity, and immune tissues (anterior kidney and spleen) [10,11,12]. These observations call for a more detailed investigation of the role of gills and pseudobranch in virus persistence and to evaluate whether both gills and pseudobranch can be useful sensors for SAV infection, regardless of PD status [13].

The pseudobranch is a reduced mandibular gill arch situated anterodorsally in the opercular cavity of a number of teleosts [14]. The pseudobranch structure can be either a free, gill-like organ fully exposed to the water as in flounder, black goby (*Gobius niger*), or a glandular organ deeply buried in the operculum tissue with fused lamellae, and no contact to the external medium as in Cyprinidae, Atlantic cod, and mature salmonids [15]. The arterial blood supply in the pseudobranch originates from the first efferent gill artery, and it splits up within the pseudobranch into a capillary system, where its efferent vessel—also known as “the ophthalmic artery”—nourishes the choroid gland of the eye [16,17]. Because of this capillary network, the pseudobranch plays roles in respiration and osmoregulation, as well as in the regulation of ocular circulation, either by controlling blood pressure in the eye or by regulating the eye fluids biochemically via the choroid gland [14]. 

The pseudobranch was previously suggested to be involved in the pathogenesis of different diseases, including, for example, parvicapsulosis (caused by *Parvicapsula pseudobranchicola*) [18] and Varracalbmi (caused by *Pasteurella* spp.) [19] in Atlantic salmon and Microsporidiosis (caused by *Loma salmonae*) in chinook salmon [20]. In advanced cases of infection with *Parvicapsula pseudobranchicola*, for example, a whitish “cheesy” material was found to obstruct the blood supply to the choroid bodies and impact the blood circulation of the eye and could thus be responsible for vision impairment in infected salmon [18]. Also, in chinook salmon infected with Loma salmonae, circulation dysfunction due to occlusion of the pseudobranchial artery by the parasitic cysts was thought to be the reason for infarction and focal necrosis of cartilage in the lower jaw of infected fish [20]. The observations that SAV could be detected in the pseudobranch tissues of both salmonids [3] and non-salmonids (flatfish) [21] upon the experimental virus challenge suggest a crucial role of that tissue in the virus pathogenesis. 

In the current work, we demonstrated the in situ localization of SAV3 in the tissues of Atlantic salmon experimentally exposed to the virus by cohabitation using an RNAscope protocol. We also compared the SAV load between the pseudobranch and the heart throughout the infection course using RT-qPCR. To investigate the role of salmon pseudobranch in the host response against SAV3 during the acute phase of infection, we compared the expression of fourteen genes involved in innate and adaptive immune activities and two mucin genes between SAV-infected and non-infected fish. Our results showed a virus tropism in the heart, pseudobranch, gills, pyloric caeca, and pancreas during the acute phase of the infection. In addition, the virus was also found in the adipose tissue associated with the internal organs of the salmon, suggesting a specific affinity of the virus to adipocytes. The inconsistent immune response of the pseudobranch against SAV3 could be responsible for the persistent low infection in that tissue and can suggest a role of the pseudobranch in SAV3 pathogenesis in Atlantic salmon.

## 2. Materials and Methods 

### 2.1. Fish

This study was approved by the Norwegian Food Safety Authority (FOTS ID: 14502), and some of the samples collected in it were part of a previously published work [22]. The post-smolt stage (average weight of 110.9 g) of Atlantic salmon (Stofnfiskur, Hafnarfjordur, Iceland; SF Optimal) was used in this study. The fish were reared at the fish facility at the Industrial and Aquatic Laboratory (ILAB, Bergen High Technology Centre, Bergen, Norway) until the challenge. The fish were unvaccinated and pre-screened at 5 g and 15 g of size for SAVs, infectious salmon anemia virus, infectious pancreatic necrosis virus, piscine myocarditis virus, piscine orthoreovirus, and salmon gill poxvirus and showed negative results for all. The parent fish were also pre-screened for all these viruses, except for SGPV, and also showed negative results.

### 2.2. SAV3 Inoculum 

The SAV3 inoculum used in this study was prepared similarly to what was previously described by Andersen et al. [23]. Briefly, heart and head kidney homogenates collected from five SAV3-infected Atlantic salmon (obtained from the Hordaland region of Norway in 2004) were pooled and used for virus propagation. The virus was propagated in the CHSE-214 cell line (ATCC^®^ CRL-1681™, Manassas, VA, USA), and the cells were grown on Leibovitz’s L-15 Medium (Lonza, Walkersville, MD, USA), supplemented with 10% fetal bovine serum (FBS; Sigma-Aldrich, Milwaukee, WI, USA) and 50 µg gentamicin mL^−1^ (Sigma-Aldrich) at 20 °C for 6 passages. Serial 10-fold dilutions of the propagated SAV3 (stock sample) were inoculated onto 24 h old CHSE-214 monolayers in 96-well plates, allowing quantification. The viral endpoint titer, measured as 50% tissue culture infective dose (TCID_50_) as described in [24], was determined to be 1 × 10^6^/mL.

### 2.3. Experimental Challenge 

To prepare SAV3 shedder fish, a total number of 45 fish were immersed in a tricaine bath (100 mg/L; Finquel® vet.; Western Chemical Inc., , Washington, DC, USA). The fish were randomly divided into three groups (I–III; 15 fish/each). Fish were immobilized and injected intraperitoneal with SAV3 inoculum using 0.2 mL of a low-dose suspension (2 × 10^2^ TCID_50_/fish; group I), a high-dose suspension (2 × 10^4^ TCID_50_/fish; group II), or virus-free Leibovitz’s L-15 cell culture medium containing 2% FBS (mock inoculum; group III). The fish with low-dose, high-dose, or SAV-free were transferred into three different 500 L seawater tanks, henceforth referred to as LD, HD, and Ctr., respectively, containing 55 cohabitant fish/each, which had been transferred to the tanks two days before the challenge started. To distinguish between shedder and cohabitant fish, all shedder fish were marked by adipose fin clipping. The shedder fish remained in the tanks throughout the entire challenge period (29 days). 

### 2.4. Management of Experimental Tanks

The experimental tanks were provided with seawater originating from 105 m depth, and the water was filtered through 20 µm drum filters and treated with UV light (135 W/m^2^). The water flow in all tanks was the same throughout the experiment, with an average flow rate of 950 L/h/tank. The water in the tank was monitored daily for temperature, salinity, and dissolved oxygen levels throughout the challenge time. During the challenge period, the LD, HD, and Ctr. tanks had dissolved oxygen saturation levels of 79–97%, 80–97%, and 79–86%, respectively; water temperature ranges of 11.7–12.3 °C, 11.7–12.3 °C, and 11.5–12.4 °C, respectively; and salinity ranges of 34.1–34.5‰, 34.1–34.5‰ and 34.2–34.5‰, respectively. All tanks had a daily photoperiod of 12:12 h light and dark, provided by an automatic artificial lighting system. During the 12 h of light, an automatic feeder dispenser fed the fish with 3 mm Nutra Olympic pellets (Skretting, Norway). The amount of food given to the fish in LD, HD, and Ctr. ranged between 56 and 140 g, 56 and 140 g, and 80 and 150 g, respectively, adjusted marginally as the fish were growing, dying, or being sampled. Clinical signs, as well as mortalities, were monitored daily in the three tanks, and dead fish were removed daily and did not undergo any further analysis. 

### 2.5. Sampling

Based on our previous experience with the SAV3 experimental challenge, fish samples were collected at 0, 7, 12, 16, 19, 20, and 29 days post-challenge (dpc). At sampling time, six cohabitant fish were randomly collected from each of the LD, HD, and Ctr. tanks at each time point. Fish were first euthanized by immersing them in a bath with an overdose of Finquel^®^ vet. 1000 mg/g (150 mg/L), and then the gross pathology was evaluated. Heart (including the valves and bulbus arteriosus) and pseudobranch samples were collected for RT-qPCR in RNAlater™ Soln. (Thermo Fisher Scientific Baltics, UAB, Vilnius, Lithuania), and samples from the heart, pseudobranch, gills, liver, spleen, posterior kidney, pyloric caeca, and pancreas were collected for in situ hybridization in 10% neutral buffered formalin.

### 2.6. RNA Isolation and cDNA Synthesis

Tissue samples in RNAlater™ were kept at −80 °C prior to RNA extraction, which was performed by adding approximately 20 mg tissue with 180 μL ATL Lysis Buffer (Qiagen^®^, Hilden, Germany) and 20 μL Proteinase K and incubation overnight at 56 °C. Extraction was performed by using QIAcube^®^ (Qiagen^®^, Hilden, Germany) with the reagents from the DNeasy Blood & Tissue Kit (Qiagen^®^, Hilden, Germany), which is supposed to copurify RNA along with DNA. DNase I (50 units/mL) was then used to remove the genomic DNA. Isolated RNA was stored at −80 °C until RT-qPCR was performed. Finally, the NanoDrop™ 2000 spectrophotometer (Thermo Scientific, Waltham, MA, USA) was used to assess the purity and yield of RNA, and samples were stored at −80 °C. Reverse transcription to synthesize cDNA was performed using 1 µg RNA input in a 20 μL reaction volume, using the QuantiTect Reverse Transcription kit (Qiagen^®^, Germany) according to the manufacturer’s instructions. After synthesis, the cDNA was diluted to prepare a working stock using nuclease-free water. Both the diluted and the original samples were stored at −20 °C until further use.

### 2.7. RT-qPCR 

#### 2.7.1. SAV Q_nsp1 Assay

The SAV3 strain was detected using the Q_nsp1 assay [25]. This broad spectrum assay detects all known SAV subtypes using primers and probe (Table 1) with final concentrations of 500 and 300 nM, respectively, and amplifies a conserved region in the 5’ end of the *nsp1* gene, giving amplicons of 107 bp. Extracted RNA was automatically pipetted by Eppendorf epMotion^®^ 5075 (Eppendorf, Hamburg, Germany) in duplicates, analyzed by RT-qPCR on an AriaMx machine (Agilent Technologies, Santa Clara, CA, USA), and evaluated with the Agilent AriaMx Real-Time PCR software (version 1.7). Each plate included a negative control sample and an inter-plate calibrator of pure SAV3 RNA, which were both run in duplicates. The cut-off quantification cycle (Cq) value was set to 40; samples with values below this Cq in duplicates were considered positive. Samples with only one positive parallel were rerun and considered positive only with positive duplicates. The template volume was 2.0 μL RNA in a total reaction volume of 20 μL, and the RT-qPCR kit used was TaqMan^®^ Fast Virus 1-Step Master Mix (Applied Biosystems®, Foster City, CA, USA). The thermal program comprised reverse transcription for 5 min at 50 °C and enzyme activation for 2 min at 95 °C, followed by 45 cycles of 15 s at 94 °C and 40 s at 60 °C. 

#### 2.7.2. Salmon Gene Expression

Pseudobranch samples (*n* = 6) were collected from fish in the LD, HD, and Ctr. tanks at 7, 12, 16, 19, 20, and 29 dpc and analyzed for the expression of different immune genes (Table 1) using RT-qPCR. Pseudobranch stock cDNA (500 ng/µL) was diluted 1:10 to analyze the expression of *IFN-α*, *IFN-γ*, *Viperin*, *Mx*, *MHC-I*, *CD8a*, *gzma*, *NK-lysin*, *MHC-II*, *CD4*, *sIgM*, *mIgM*, and *EF1α*, whereas 1:3 dilution was used to analyze *sIgT-B*, *mIgT-B, Muc-2*, and *Muc-5*. The expression was also compared between gills and pseudobranch collected from the control fish (0 dpc) in the same cDNA amounts. The RT-qPCR experiments were performed using the ABI 7900HT fast instrument (Thermo Fisher Scientific, Inc., Waltham, Massachusetts, USA) and Brilliant III Ultra-Fast SYBR^®^ Green QPCR Master Mix (Agilent Technologies, Palo Alto, CA, USA; Cat. No. 600882). Each sample was analyzed in duplicates, using a total reaction mix volume of 20 µL per well (2 µL cDNA, 0.8 µL [400 nM] of each forward and reverse primers, 10 µL of the master mix, 0.3 µL [300 nM] of ROX reference dye, and 6.1 µL of nuclease-free water). The elongation factor 1α (*EF1α*) gene was used as a reference gene, and non-template wells were run on each plate as a negative control. The following thermocycler conditions were used: initial denaturation (3 min at 95 °C) followed by 40 cycles of denaturation (5 s at 95 °C) and annealing (15 s at 60 °C). The extension was performed for 5 s at 55 °C. Finally, a melting curve was made by measuring the fluorescence during a temperature range of (60–95 °C) to confirm the specificity of the end-product amplicon in the reaction. Fluorescence was measured, expressed as relative fluorescence units (RFUs) and quantification cycles (Cq) for every reaction that was measured. All gene expression values (Cq values) were normalized to *EF1α* values [26], and the relative expression of target genes was calculated using the ΔΔ *C*_t_ method [27].

### 2.8. In Situ Hybridization (ISH)

We demonstrated the SAV3 localization in situ in tissues of infected fish to study the tissue tropism of the virus. Formalin-fixed paraffin-embedded (FFPE) sections from the heart, pseudobranch, gills, liver, spleen, posterior kidney, pyloric caeca end pancreas collected from fish in the HD and Ctr. groups at 7, 12, 16, 20, and 29 dpc, were prepared. A protocol based on RNAscope technology was applied using RNAscope^®^ 2.0 HD Red Chromogenic Reagent Kit (Advanced Cell Diagnostics Inc., Newark, CA, USA). In this protocol, paired double-Z oligonucleotide probes targeting a SAV3 nucleotide stretch (region 46 to 944 bp) of the genomic RNA of Norwegian SAV isolate Hav1 (accession number: GenBank AY604235.1) were used. A negative control probe targeting the Bacillus subtilis SMY strain gene DapB (accession number EF191515, region 414 to 862 bp; Cat. No. 310043, Advanced Cell Diagnostics) was used to subtract the background signals, and a positive control probe derived from the peptidylprolyl isomerase B (PPIB) gene of Atlantic salmon (accession number NM_001140870.2, targeting region 20 to 934 bp; Cat. No. 494421, Advanced Cell Diagnostics) was applied to confirm both the mRNA integrity in the samples and the functionality of the ISH experiment. RNAscope was performed following the manufacturer’s instructions. Briefly, FFPE sections were deparaffinized in xylene and rehydrated through a series of alcohol washes. The rehydrated sections were then treated with hydrogen peroxide at room temperature for 10 min to block endogenous peroxidases. Then, the sections were boiled in a target retrieval buffer for 15 min and incubated with protease at 40 °C for 15 min. The boiled sections were hybridized with the probes specified earlier at 40 °C for 2 h and then run through a sequence of signal amplification (40 °C for 15 or 30 min) and washing steps. Finally, the hybridization signal was visualized using Fast Red stain, and all slides were counterstained using Mayer’s hematoxylin (Chemi Teknikk, 5B-535) diluted in distilled water 1:1 (*vol/vol*) for 2 min. Sequential and adjacent pseudobranch sections to those analyzed in ISH were stained with H&E [41]. All slides were examined with an Olympus BX51 microscope (Olympus) and scanned using a NanoZoomer S210 digital slide scanner (Hamamatsu Photonics KK, Shizuoka, Japan). The resulting scanned images were visualized using viewer software (NDP.view2 U12388-01; version 2.7.25, Hamamatsu Photonics).

### 2.9. Statistics

Because data were not normally distributed and different groups had unequal variances, nonparametric statistical analyses were performed using JMP software (JMP^®^, Version 11, SAS Institute Inc., Cary, NC, USA, 1989–2007) with α value set to 0.05. While the Mann–Whitney U test was conducted to detect statistically significant differences between the *Muc-2* and *Muc-5* Cq values between the pseudobranch and the gills, the significance of the difference in the immune gene expression between the virus-challenged and control groups was determined for each gene at each time point using Kruskal–Wallis test followed by Dunn’s multiple comparison test. GraphPad Prism version 6.0 (San Diego, CA, USA) was used to make graphs.

## 3. Results 

### 3.1. In Situ Hybridization 

**Figure 1:**
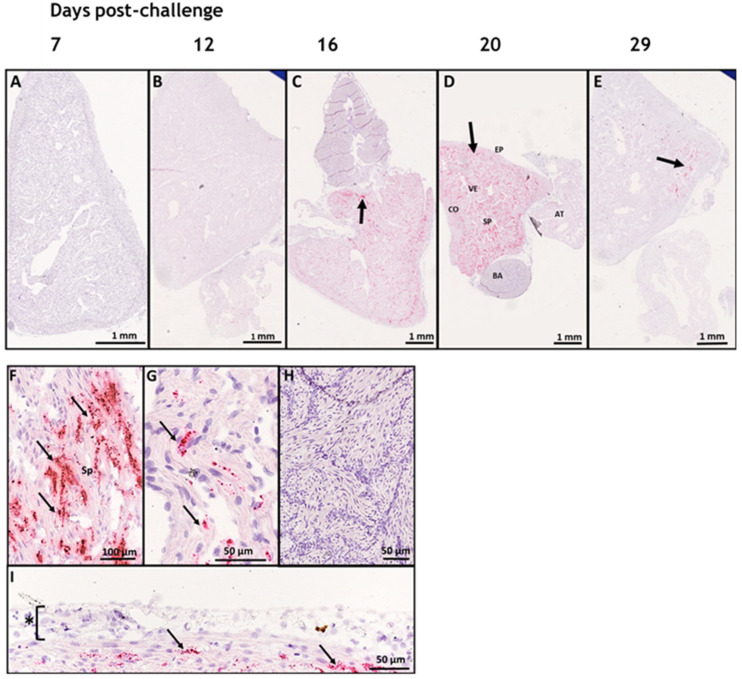
SAV3 invaded heart ventricles extensively during the current cohabitation challenge. In situ hybridization (RNAscope) of SAV3 in the heart of Atlantic salmon challenged with the virus by the cohabitation method. Heart tissues collected from fish in the high-dose group at 7 (**A**), 12 (**B**), 16 (**C**), 20 (**D**), and 29 (**E**) days post-challenge showed SAV-specific hybridization signals (red staining and arrows). The different heart compartments (epicardium [EP], ventricle [VE], atrium [AT], compact [CO] and spongy [SP] myocardial layers, and bulbus arteriosus [BA]) in (D) are shown at a higher magnification in (**F**–**I**). SAV-specific signal (arrows) was detected in the spongy myocardium of ventricle (**F**) and atrium (**G**) and compact myocardium of ventricle (**I**). No SAV-specific signal has been detected in bulbus arteriosus (**H**) or the epicardium (* in (**I**)).

**Figure 2:**
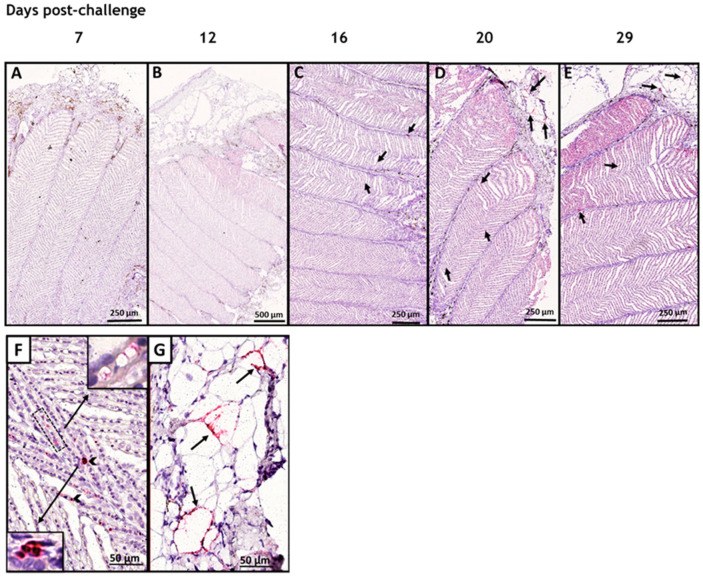
SAV3 infected Atlantic salmon pseudobranch during the acute phase of the infection. In situ hybridization (RNAscope) of SAV3 in the pseudobranch of Atlantic salmon challenged with the virus by the cohabitation method. Pseudobranch tissues collected from fish in the high-dose group at 7 (**A**), 12 (**B**), 16 (**C**), 20 (**D**), and 29 (**E**) days post-challenge showed SAV-specific hybridization signal (red staining and arrows) in (**C**–**E**). The SAV-specific staining detected in (**D**) is shown at a higher magnification in (**F**,**G**). SAV hybridization was associated with the luminal membranes of endothelial/pillar cells (dotted frame box in (**F**)), with the cytoplasm of irregular cells at the base of the lamellae (arrowhead in (**F**)), and with adipocyte cytoplasm in adipose tissue around the pseudobranch (arrows in (**G**)).

**Figure 3:**
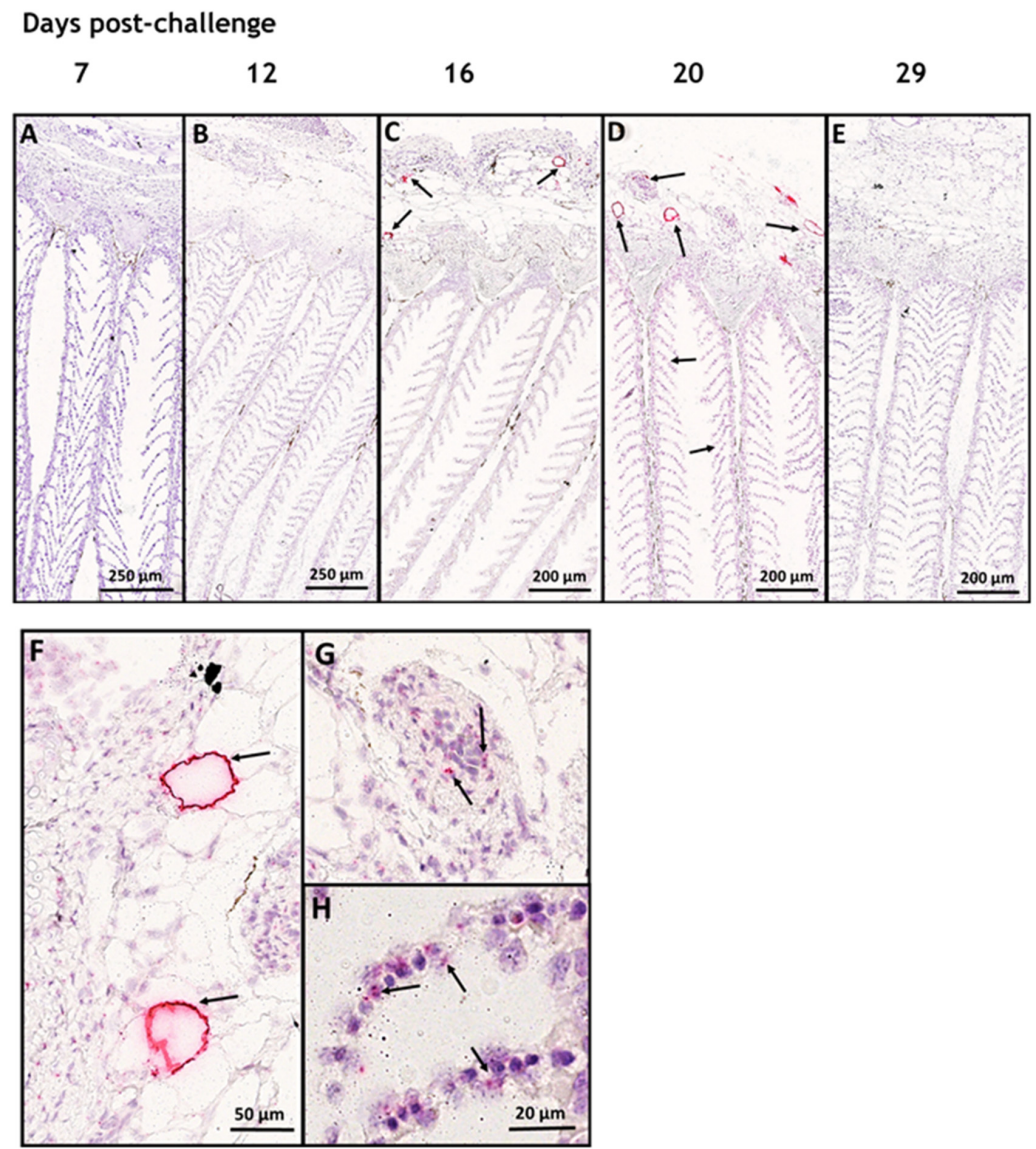
SAV3 affected gill-associated adipose tissue more than gill lamellae. In situ hybridization (RNAscope) of SAV3 in gills of Atlantic salmon challenged with the virus by the cohabitation method. Gill tissues collected from fish in the high-dose group at 7 (**A**), 12 (**B**), 16 (**C**), 20 (**D**), and 29 (**E**) days post-challenge showed SAV-specific hybridization signal (red staining and arrows) in (**C**,**D**). SAV-specific staining detected in (**D**) is shown at a higher magnification in (**F**–**H**) to show the hybridization associated with the cytoplasm of adipocytes in gill-associated adipose tissue (arrows in (**F**)), in a few cells in the gill-associated blood vessels (arrows in (**G**)), and in some cells running through the vascular space of the secondary lamella (arrows in (**H**)).

**Figure 4:**
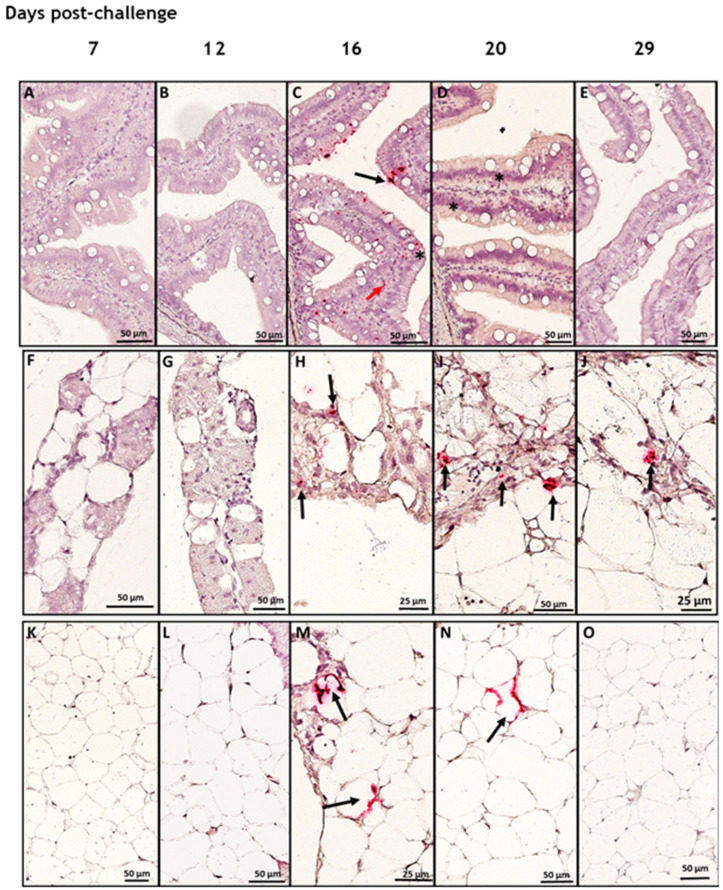
The pyloric ceca/pancreas structure with the surrounding fat layer in Atlantic salmon was infected with SAV3 during the acute phase of the current cohabitation infection challenge. In situ hybridization (RNAscope) of SAV3 in pyloric caeca (**A**–**E**), pancreas tissue (**F**–**J**), and their associated adipose tissue (**K**–**O**) of Atlantic salmon challenged with the virus by the cohabitation method. Tissues collected from fish in the high-dose group at 7 (**A**,**F**,**K**), 12 (**B**,**G**,**L**), 16 (**C**,**H**,**M**), 20 (**D**,**I**,**N**), and 29 (**E**,**J**,**O**) days post-challenge, showed SAV-specific hybridization signal (red staining and arrows) in the PC at 16 and 20 dpc on the apical part of the villi (probably attached to the mucus; black arrows), in the epithelial columnar cells (asterisks), and cells in the lamina propria (red arrows). SAV-specific staining was also detected in some cells in the pancreas region at 12, 16, and 20 dpc (black arrows in (**H**, **I**, and **J**), respectively) and in the cytoplasm of adipocytes in the associated adipose tissue at 12 and 20 dpc (black arrows in **M** and **N**, respectively).

**Figure 5:**
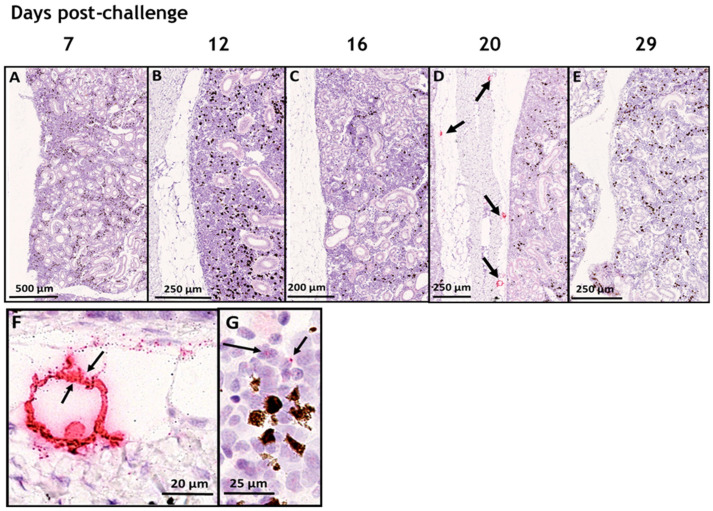
The Atlantic salmon posterior kidney was of low susceptibility to SAV3 during the acute phase of the current cohabitation infection. In situ hybridization (RNAscope) of SAV3 in the posterior kidney of Atlantic salmon challenged with the virus by the cohabitation method. Posterior kidney tissues collected from fish in the high-dose group at 7 (**A**), 12 (**B**), 16 (**C**), 20 (**D**), and 29 (**E**) days post-challenge showed SAV-specific hybridization signal (red staining and arrows) in (**D**). SAV-specific staining detected in (**D**) is shown at a higher magnification in (**F**,**G**) to show the hybridization associated with the cytoplasm of adipocytes in renal-associate adipose tissue (arrows in (**F**)) and in association with few cells in the parenchyma (arrows in (**G**)).

**Figure 6:**
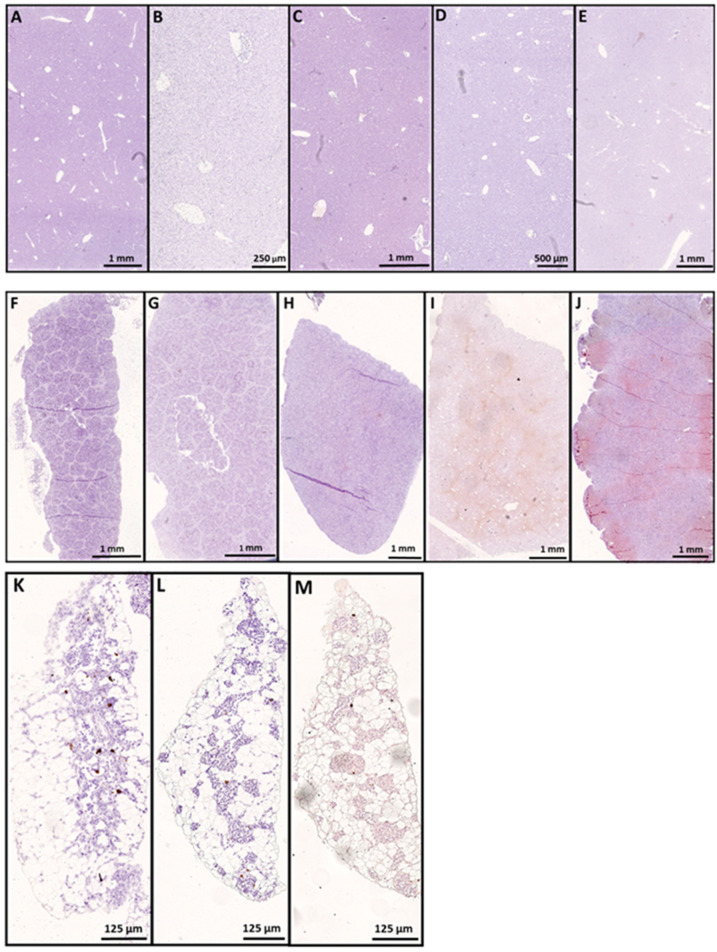
The liver and spleen with their surrounding fatty layer were insusceptible to SAV3 infection in the current cohabitation model. In situ hybridization (RNAscope) of SAV3 in the liver (**A**–**E**), spleen (**F**–**J**), and its associated adipose tissue (**K**–**M**) of Atlantic salmon challenged with the virus by the cohabitation method. Tissues collected from fish in the high-dose group at 7 (**A**,**F**,**K**), 12 (**B**,**G**), 16 (**C**,**H**), 20 (**D**,**I**,**L**), and 29 (**E**,**J**,**M**) dpc, showed no SAV-specific hybridization signal at any of the time points analyzed.

We studied the tropism of SAV3 in situ in tissues collected from cohabitant fish exposed to the high dose of the virus at different time points after the challenge, using RNAscope protocol. Our results showed no SAV3-specific signal detected in tissues analyzed before 16 dpc. Heart, pseudobranch, and pancreas showed SAV3-specific hybridization at 16, 20, and 29 dpc (Figure 1 and Figure 2). Tissues from the gills, pyloric caeca, and its surrounding adipose tissue had the virus particles at 16 and 20 dpc (Figure 3 and Figure 4). The virus was detected on a single occasion in the posterior kidney after 20 days of cohabitation (Figure 5). Neither the liver nor the spleen with its surrounding adipose tissue showed any SAV3-specific hybridization at any of the time points (Figure 6). 

The heart tissue showed SAV3-specific hybridization at 16, 20, and 29 dpc, where the signal seems to dominate the ventricle compartment (mostly in the muscle cells of both the compact and spongy myocardium layers of the heart (Figure 1F)) as compared to the atrium (Figure 1G). However, neither the bulbus arteriosus nor the epicardium showed any SAV3-specific signal (Figure 1H,I).

In the pseudobranch, at 20 dpc, the signal predominates intravascularly and seemingly in tissue compartments where no circulating cells are occupying the available space (Figure 2D,F). Furthermore, while the signal appears to stick to the luminal membranes of several endothelial/pillar cells, other populations of larger and irregular cells at the base of the lamellae seem to show an intracytoplasmic signal (Figure 2F). 

Interestingly, SAV3-specific hybridization could also be detected in the cytoplasm of adipocytes in the adipose tissue surrounding the pseudobranch (Figure 2G). Pseudobranch sections prepared serially to those studied by RNAscope, stained with H&E, and examined for any histopathological changes that might have been caused due to SAV exposure showed no significant histopathological alterations in areas adjacent to those where the SAV3-specific hybridization signals were detected on the RNAscope sections (Supplementary Figure S1). 

As for the hybridization results of SAV3-specific probes in the gills, the signal was consistently detected in the cytoplasm of adipocytes in the gill-associated adipose tissue (Figure 3C,D,F). In addition, a few of the cells running through the vascular space of the secondary lamella and in the blood vessels associated with the gills also showed a positive signal for SAV3 (Figure 3G,H). The SAV3-specific signal identified in the gill lamella was, however, less prominent as compared to that of the pseudobranch. 

The SAV3-specific probes were also hybridized in the pyloric caeca and the pancreas, showing varied degrees of positivity between 16 and 29 days post-challenge (Figure 4A–O). In the pyloric caeca, the SAV was detected in association with the columnar epithelial cells and in the mucus layer on its apical surface, as well as in some cells harbored in the lamina propria of the intestinal mucosa (Figure 4C,D). Rare positive cells could also be detected in association with the pancreatic tissue (Figure 4H–J). As in the other tissue, the SAV3-specific signal was also detected in the cytoplasm of adipocytes surrounding the pancreas (Figure 4M,N). 

In the trunk kidney, the detection of SAV3 was only observed at 20 dpc with a ‘humble’ hybridization staining (Figure 5D). At that time point, the signal seemed to be associated with the cytoplasm of adipocytes in the surrounding adipose tissue (Figure 5F), as well as with a few cells dispersed in the renal parenchyma (Figure 5G).

Nevertheless, the other compartments of the trunk kidney, including proximal and distal tubules, and the renal corpuscle showed no SAV-specific hybridization at any of the time points analyzed. Interestingly, the liver, spleen, and the adipose tissue surrounding the spleen showed no SAV hybridization at any of the time points (Figure 6A–E), (Figure 6F–J) and (Figure 6K–M), respectively.

### 3.2. SAV-nsp1 Quantification 

**Figure 7:**
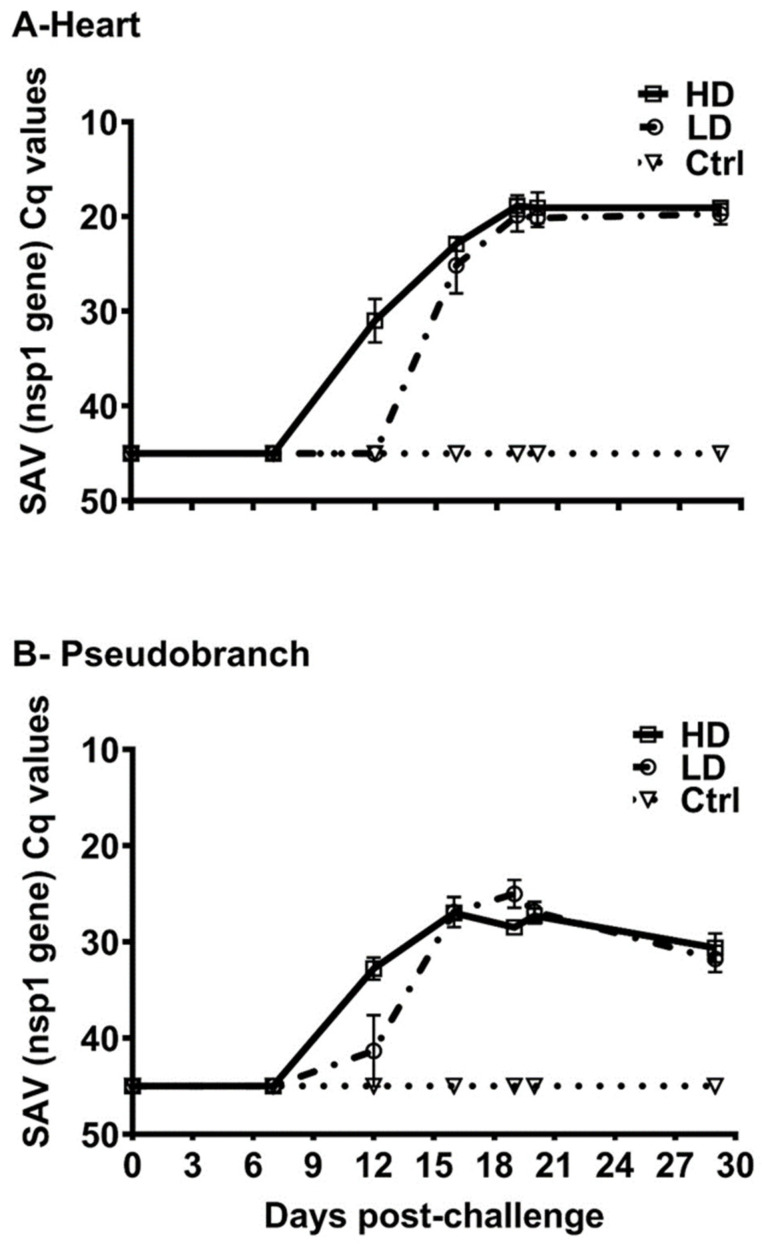
SAV3 caused a persistent infection in the pseudobranch with a lower virus load than in the heart. SAV3 infection load in (**A**) heart and (**B**) pseudobranch of salmon fish exposed to no (Ctr.; control fish) low (LD) or high (HD) dose of the virus (challenge by cohabitation with virus-shedding fish) from 0 to 29 dpc based on the expression of SAV-*nsp1* gene (Ct values; median with range) using RT-qPCR (Q_nsp1 assay).

The SAV load in the heart and pseudobranch tissues was studied throughout the challenge period (0–29 dpc) by quantifying the expression of the *nsp1* gene in both tissues from fish in the LD, HD, and Ctr. tanks using quantitative RT-qPCR and the *Q_nsp1* assay. The heart and pseudobranch of fish exposed to low and high doses of SAV showed no *nsp1* expression (median Cq value > 44) at 7 dpc. At 12 dpc, fish in the HD group showed higher SAV Cq values than those in the LD group, with *nsp1* median Cq values of 31 vs. 45 in the heart and 28 vs. 41 in the pseudobranch, as shown in Figure 7. The difference in expression between the LD and HD groups was less pronounced at the later time points. At 16 dpc, for example, while fish in LD and HD groups had comparable levels of *nsp1* expression in the heart (Cq values of 23 and 24, respectively), both groups showed higher median Cq values (27 for both) in the pseudobranch. While the virus peak in the heart was detected at 19 dpc in both LD (median Cq = 18.89) and HD (median Cq = 20.64) groups, the peak of the virus in the pseudobranch was detected at 16 dpc for fish in the HD group (median Cq = 27.1) and at 19 dpc for fish in the LD group (median Cq = 25.86). The virus load remained relatively constant in both heart and pseudobranch tissue samples toward the end of the experiment (Figure 7), with less virus in the pseudobranch (median Cq of 31.61 and 30.21, for LD and HD, respectively) as compared to the heart (median Cq of 20.30 and 18.84, for LD and HD, respectively) at 29 dpc. Fish in the control group showed no nsp1 gene expression at any of the time points analyzed (Figure 7).

### 3.3. Immune Response in Pseudobranch against SAV

We investigated the immune response in the pseudobranch to the challenge with SAV3 by analyzing the expression of genes related to both innate and adaptive immune mechanisms. 

#### 3.3.1. Genes Linked to Antiviral Activity 

**Figure 8:**
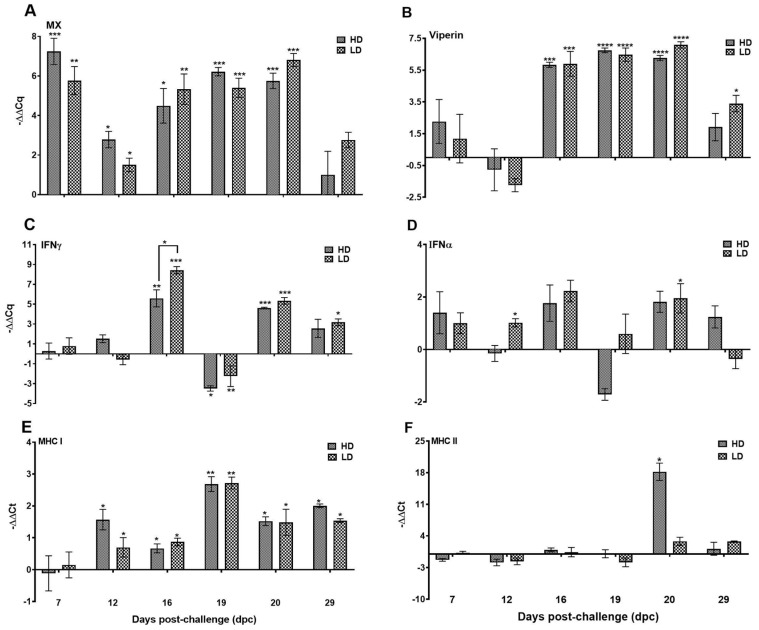
An innate immune response was mounted in the Atlantic salmon pseudobranch to SAV3 infection in the current cohabitation challenge model. Relative gene expression (median with range) of (**A**) myxovirus resistance 1 (*Mx*), (**B**) virus inhibitory protein, endoplasmic reticulum-associated, interferon-inducible (*viperin*), (**C**) interferon-gamma (*IFN-γ*), (**D**) interferon-alpha (*IFN-α*), (**E**) major histocompatibility complex class I (*MHC-I*), and (**F**) major histocompatibility complex class II (*MHC-II*), in the pseudobranch of Atlantic salmon exposed to low (LD) and high (HD) doses of SAV from 0 to 29 dpc. Asterisks above solid lines between groups represent significant changes between the denoted groups, and asterisks above the individual groups represent a significant difference between these groups and the non-exposed control fish. * *p* < 0.05, ** *p* < 0.005, *** *p* < 0.0005 and **** *p* < 0.0001.

Genes related to the antiviral activity showed a generic upregulation pattern in the pseudobranch in response to SAV exposure. As early as 7 dpc, both LD and HD groups showed high transcript levels of the *Mx* gene that were significantly different from that of the control group and which lasted up to 20 dpc (Figure 8A). The change in expression of *viperin* and *IFNγ*, on the other hand, showed a late response to virus exposure and was not significant in the SAV3-challenged groups until 16 dpc. Between 16 and 20 dpc, the mRNA of *Viperin* showed a steady high level in the virus-exposed groups (median ≥ 5.5-fold) before it dropped toward the end of the experiment, leading to transcript levels only significant in the LD group (Figure 8B). The SAV-infected fish in LD and HD groups also showed a generally higher *IFNγ* transcript (median ≥ 3-fold) from 16 dpc and until the end of the experiment with a transient downregulation (median < 3.5-fold) at 19 dpc in both groups (Figure 8C). Interestingly, *IFNα* showed a general upregulation throughout the infection course; however, the change was only significant for the LD group at 12 and 20 dpc (Figure 8D). 

#### 3.3.2. Major Histocompatibility Molecules

The change in *MHC-I* transcript levels was consistent, and a general upregulation with varied fold numbers (median ≥ 0.9) was detected throughout the infection course in both LD and HD groups; however, the expression was not significantly different from that of the control group until 12 dpc (Figure 8E). On the other hand, the analysis of the difference in *MHC-II* expression in the pseudobranch between the SAV3-challenged and control fish showed a single transient, yet prominent, significant value in fish in the HD group around the virus peak in the pseudobranch (RT-qPCR) as shown in Figure 8F.

#### 3.3.3. Genes Linked to T Cells

**Figure 9:**
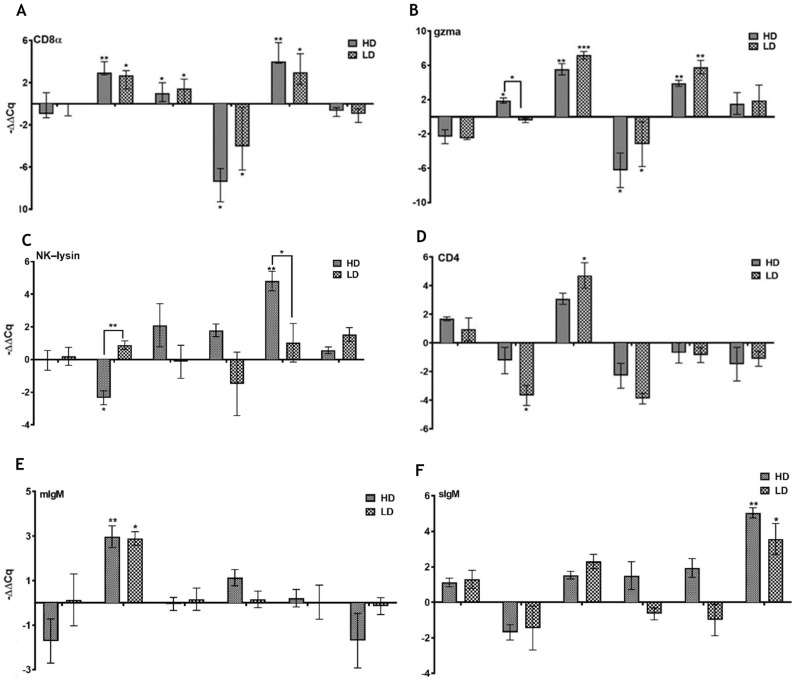

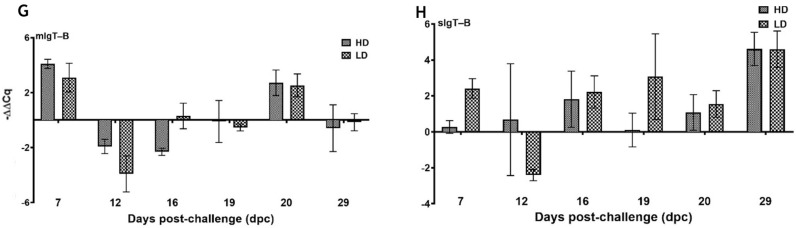
SAV3 infection provoked an inconsistent adaptive immune response in Atlantic salmon pseudobranch in the current virus cohabitation challenge model. Relative gene expression (median with range) of (**A**) cluster of differentiation 8 alpha (*CD8a*), (**B**) granzyme A (*gzma*), (**C**) natural killer lysin (*NK–lysin*), (**D**) cluster of differentiation 4 (*CD4*), (**E**,**F**) membrane and secretory immunoglobulin M (*mIgM* and *sIgM*, respectively), and (**G**,**H**) membrane and secretory immunoglobulin T sub-isotype B (*mIgT–B* and *sIgT–B*, respectively) in pseudobranch of Atlantic salmon fish exposed to low (LD) and high (HD) doses of SAV from 0 to 29 dpc. Asterisks above the groups and the solid lines represent significant differences between the denoted group from the non-exposed control fish and the LD and HD, respectively. * *p* < 0.05, ** *p* < 0.005 and *** *p* < 0.0005.

The expression patterns of *CD8a* and granzyme A (*gzma*) corresponded to each other in the pseudobranch of fish exposed to SAV3. The mRNA levels of both genes were significantly higher (median of 1.5–7-fold) in the virus-exposed groups in the period between 12 and 20 dpc (as compared to the control), with an exception at 19 dpc, where a significant downregulation of both genes (median ≥ 2.5-fold) was recorded (Figure 9A,B). NK-lysin showed a different pattern of expression from that of *CD8a* and *gzma*. While fish in the LD group had no significant differences from that of the control group along the SAV infection course, fish in the HD group showed a significantly low level (median = 2.5-fold) at 12 dpc and a significantly high level (median = 5-fold) at 20 dpc as compared to both the control and the LD groups (Figure 9C). As for the *CD4*, there were no significant differences in the mRNA levels between the SAV3-infected and control fish, except for the lower and higher transcripts in the LD group at 12 and 16 dpc, respectively (Figure 9D). 

#### 3.3.4. Immunoglobulins 

After SAV exposure, genes related to adaptive humoral immune response (immunoglobulin genes) showed a less prominent change as compared to that of genes linked to CD8 T cells or the antiviral activity. For IgM, while the mRNA level of *mIgM* in LD and HD groups showed a single significant upregulation event (median ≥ 3-fold) at 12 dpc, the change in the sIgM was remarkable at a later time (29 dpc; median ≥ 3.5-fold higher in virus-exposed groups), as shown in Figure 9E,F. On the other hand, the expression patterns of *mIgTB* and *sIgTB* in the challenged groups showed no significant differences from that of the control group at any of the time points analyzed (Figure 9G,H). 

### 3.4. Mucin Gene Expression

**Figure 10:**
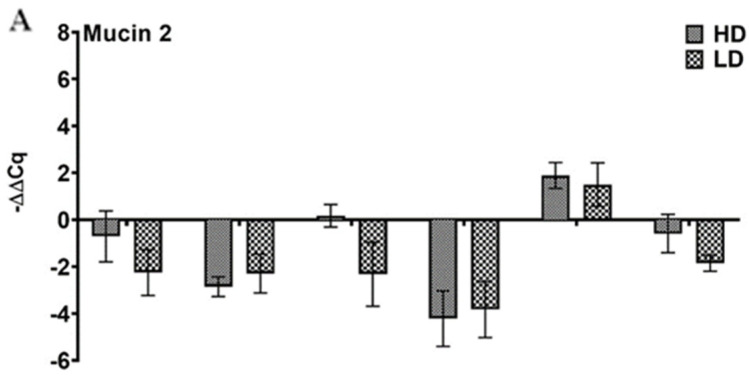

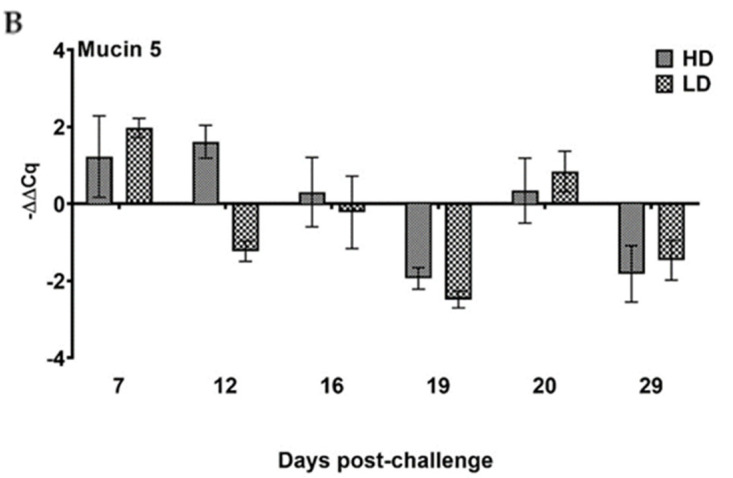
Atlantic salmon pseudobranch mucin gene expression did not change upon SAV3 infection in the cohabitation model. Relative gene expression (median with range) of mucin genes (**A**) *Muc-2* and (**B**) *Muc-5* in the pseudobranch of Atlantic salmon fish exposed to low (LD) and high (HD) doses of SAV from 0 to 29 dpc showing no significant change at any of the time points analyzed.

We also studied the change in the mRNA levels of two mucin genes (*Muc-2* and *Muc-5*; in the pseudobranch after SAV3 exposure. Although the varied mRNA amounts measured throughout the infection trials showed a general tendency of downregulation, the SAV3-infected fish pseudobranch showed no significant difference from the control fish at any of the time points analyzed (Figure 10).

## 4. Discussion

In this study, an in situ hybridization protocol (RNAscope) was successfully used to detect SAV3 in situ in tissues of the virus-infected salmon and to demonstrate the tropism of the virus during the acute phase of the disease by employing a challenge model based on cohabitation with virus-shedding fish. In addition, we also described the response of selected immune genes to SAV3 infection in the pseudobranch, a tissue that was claimed to harbor the virus for a long time regardless of the state of the disease it causes [13]. Our analyses avoided the dead fish and depended only on fish that had been euthanized at the time of sampling, and there could be some samples with different virus distribution and immune response patterns that were not studied. 

When PD was first characterized in salmon, the injection of spleen homogenate from infected fish into healthy individuals could reproduce the infection [42,43,44], and the virus was suggested to localize in the infected spleen. However, although varied degrees of gross and microscopic changes were detected in the spleen and liver of SAV3-infected fish in natural [45] and experimental [3,46] cases, there was no mention that the virus was detected in these tissues in these studies. In our study, the virus was not detected in the spleen or liver of the SAV3-infected fish, not even around the virus peak. In the posterior kidney, the virus was detected in a few cells dispersed in the renal parenchyma and was absent in the excretory compartments. Given that SAV3 can be detected in the blood of the virus-infected fish during the acute phase of infection [47], the humble detection of the virus in the well-perfused tissues in our study becomes interesting. Indeed, the time interval of the acute phase of SA3 infection (when the viremia is to be expected) in a previous challenge trial was estimated to be 3–5 weeks post-challenge [3]. However, in that study, the virus was introduced to the fish through the i.p. route with an expected higher infection load and quicker dissemination to the blood than in our cohabitation model.

The time of the detection of SAV3 RNA in a few cells in the pancreas between 16 and 29 dpc in our model agreed with the significant detection of the virus in the pancreatic tissue 21–37 days post-injection in the i.p. challenge model [3]. However, in that study, the pancreas had the lowest prevalence of SAV3 RNA among the other salmon tissues and was proposed to be an unsuitable tissue for PD diagnosis using RT-PCR. In addition, the pancreas samples analyzed in that study were combined with the pyloric caeca sample and did not necessarily represent the pancreas results exclusively. In our challenge model, the virus detection in the pyloric caeca was confirmed at 16 and 20 dpc in association with the cells wandering in the lamina propria of the intestinal mucosa and also in columnar epithelial cells and the covering mucus layer. These results agree with the previous findings showing SAV-infected fish shedding the virus in feces during experimental challenge [48].

In their tropism study, Andersen et al. [2] showed that the pseudobranch and heart ventricle had the highest prevalence of viral RNA regardless of PD status. In our results, both heart and pseudobranch showed a consistent detection of SAV3 in the infected fish from the time of the virus peak until the end of the experiment (RNAscope and RT-qPCR data). Nevertheless, although the RNAscope data showed no SAV detection before 16 dpc, the early onset of the ‘humble’ detection of the virus (Cq = 37) in the pseudobranch of fish before the heart in the LD group, using the highly sensitive Q_nsp1 assay, can suggest the SAV kinetic direction between the two tissues to be from outward (pseudobranch) to inward (heart). Indeed, the detection of SAV in the pseudobranch of salmonids [2] and non-salmonid fish [19] in the virus challenge experiments affirms the importance of that tissue for SAV pathogenicity. However, in contrast to the significant heart lesions in Atlantic salmon due to SAV3 infection [49], the potential role in pathogenicity was not necessarily accompanied by SAV-specific histological alterations in the pseudobranch, the results that agree with the finding of Christie et al. [9], where no histopathological alteration was detected in the pseudobranch of SAV3-infected Atlantic salmon. The absence of histopathology in the pseudobranch can probably be attributed to, among many other factors, the low virus load in the pseudobranch. Although this line of argument can be supported by the previous results showing the severity of PD pathology in salmon to correlate positively with the virus amount [31], it is still interesting that other tissues with decent SAV amounts (e.g., liver) can show pathological alterations in SAV-infected fish [3,21].

Besides the virus detection in the lamellae of the pseudobranch, the virus was also detected in the tissue-associated adipocytes. Indeed, there was a consistent pattern of detecting SAV in the organs-associated adipose tissue in salmon, an observation that suggests a virus-specific affinity to one or more of the components in the salmon adipocytes. Viable SAV particles were identified in the lipid fraction leaking from the dead PD-infected salmon and were proposed to contribute to the horizontal transmission of the virus, at least in vitro [50]. A significant amount of literature also showed the susceptibility of mammalian adipocytes to different viruses, including, for example, Ebola [51] and COVID-19 virus [52], and several laboratory studies reported that alphavirus infections in obese mice were more severe as compared to healthy-weight animals [53]. The fact that adipocytes differentiate from fibroblasts [54] (known target cells for alphavirus replication [55]) makes them also a potential candidate for alphavirus propagation. Interestingly, the adipose tissue associated with the spleen showed no SAV detection, probably due to a difference in the cellular and/or molecular components between the different adipose tissue sites. 

Despite the SAV detection in the adipose tissues associated with both the pseudobranch and the gill tissues, the virus detected in the functional compartment of the gills (i.e., lamella) was less prominent than that of the pseudobranch. This observation agrees to a certain extent with the finding that the pseudobranch can have a SAV3 prevalence percentage higher than that of the gills in virus-challenged fish up to 91 dpc [3]. The low prevalence of SAV in gills can be linked to the pathogen clearance ability of the gills exerted by their continuously secreted mucus [56]. Such a feature has not been confirmed yet in the salmon pseudobranch, considering its deeply buried nature, the opercula tissue, and its fused lamellae, with no contact with the aquatic milieu [15] or expectation of mucus secretion. An argument that can be supported at least by our results showing no change in the low mRNA levels of two mucin genes (*Muc-2* and *Muc-5*, previously studied in Atlantic salmon gills and showed expression changes upon pathogenic insults [39]) in the pseudobranch of SAV3-infected fish. Despite the potential difference, both the gills and pseudobranch tissues were stated in the previous literature to harbor SAV for a long time without noticeable pathological damage [57].

The change in the immune components of the pseudobranch in response to the persistent infection was interesting to investigate. Among the innate immune genes regulated in the pseudobranch after SAV exposure, *Mx* gene was the earliest and the most consistent throughout the infection course. Mx proteins are known for their antiviral activities in different species [58], with either an interferon-dependent [59] or independent [60] induction mechanism in different viral infections. The latter kind of induction is more likely the one responsible for the early upregulation of *Mx* in this study since it was noticed regardless of the regulation state of *IFN* genes. However, a positive correlation was established between the IFN-dependent stimulation of Mx and the protection against SAV-induced cytopathogenic effects in vitro [61,62], and hence, the efficacy of the response becomes questionable. The expression of the other antiviral genes in this study (*viperin*) seemed to be *IFN*-dependent, showing late upregulation that coincided with the upregulation of *IFN-γ* around the virus peak. In mammals, interferons (including *IFNα*, *IFNβ*, and *IFNγ*) can induce an antiviral state in both virus-infected and uninfected cells [63]. In this study, the absence of a consistent increase in *IFN-α* in the SAV-infected pseudobranch along the infection course can suggest a reduced antiviral activity against the virus, probably contributing to the persistent infection of the SAV in that tissue. On the other hand, the increase in *IFN-γ* around the virus peak does not necessarily mean efficient protection against SAV, considering the results of the in vitro infection model showing rIFN-γ to have mild antiviral activity against SAV3 [64]. In all cases, the early initiation of the IFN system as part of the host innate immune response in salmon was considered important for protection against SAV3 [49].

Genes related to the adaptive immune response showed a modest change upon SAV3 infection. The response of the two T-cell co-receptors (CD4 and CD8, which enhance the recognition of the MHC–peptide complex by the T-cell receptor and hence the T-cell activation [65]) to SAV infection showed different patterns. We confirmed an increase in the transcription of the cytotoxic T-cell marker (*CD8a*) and its killing mediator (*gzma*) in the SAV3-infected pseudobranch. Both molecules are associated with adaptive immune cytotoxic T-cells and are involved in intracellular antigen recognition and cell-mediated killing, respectively, and their increase in response to SAV infection is probably to clear the virus-infected cells. The consistent upregulation of the *CD8a* and *gzma*, as well as *MHC-I* in the pseudobranch of SAV3-infected fish, is an indication of mounting a Th1 cytotoxic immune response against SAV, supporting a previous proposal of the implication of the adaptive cell-mediated immunity in SAV-infected salmon in the in vivo infection model [49]. Accordingly, the upregulated *MHC-II* (expressed by the professional antigen-presenting cells; APCs [66]) at a later time point suggests kinetics of the APCs in pseudobranch in response to the exogenous virus material released from the SAV-infected cells cleared by the Th1 mounted response. As the presentation of pathogen-derived antigens by *MHC-II* on the APCs’ surfaces is essential to the elicitation of CD4 T-cell binding [66], a simultaneous increase in the *CD4* expression (due to CD4 cell kinetics) could be expected. Interestingly, this was not the case where the only *CD4* upregulation event was identified around the virus peak. The discontinuation of T-cell responses in the pseudobranch toward the end of the SAV3 infection course can suggest an insufficient T-cell-derived defense against the virus in that tissue.

The upregulation of *mIgM* around the virus peak in the pseudobranch suggests the kinetics of B cells in response to the infection. A reaction that can be seen as part of the B cell conserved antigen recognition and processing [67], even earlier than the other professional APCs (as judged by late expression of *MHC-II*). If not for the unchanged expression of *MHC- II*, the synchronized upregulation of *CD4* at 16 dpc and *mIgM* at 12 dpc (hence the kinetics of T helper and B cells, respectively) could have been related to the late upregulation of *sIgM*. However, a T-cell-independent activation of B cells, previously studied in vesicular stomatitis virus [68], can be suggested to be responsible for the late increase in *sIgM*. The upregulation of *sIgM* can indicate a possibility of producing SAV3-specific antibodies. This suggestion can be supported by the results showing the detection of SAV-specific antibodies in the plasma of SAV-challenged fish at 3–6 weeks post-challenge [69]. Interestingly, the *IgT* pattern of expression was different from that of the IgM, where no response was detected. Indeed, the mucosal tissue is the main compartment of teleosts’ IgT isotype [70], a feature that has not been used to describe the salmon pseudobranch so far. With the no change pattern in *IgT* expression, the importance of that isotype for the salmon pseudobranch is probably trivial.

## 5. Conclusions

We managed to use an in situ hybridization protocol (RNAscope^®^) to detect SAV3 in situ in tissues of the virus-infected salmon and demonstrated the tropism of the virus during the acute phase of the experimental cohabitation infection. While no SAV3 was detected in the spleen or liver, the heart, gills, pseudobranch, pyloric caeca, posterior kidney, and pancreas showed varied levels of virus detection. Interestingly, we also demonstrated a ubiquitous identification of the virus in the adipose tissue distributed around the different internal organs in salmon, except for that around the spleen. The persistent low infection of the virus in the pseudobranch, along with the inconsistent immune response to SAV3 infection, can suggest a pivotal role of the pseudobranch in SAV3 pathogenesis in Atlantic salmon.

## Figures and Tables

**Table 1:** Sequences of oligonucleotide primers used in real-time PCR.

Gene Name	Primer Sequences (5′-3′)	Reference	Product Size	Accession Number
*Interferon alpha (IFN-α)*	F: TGCAGTATGCAGAGCGTGTG R: TCTCCTCCCATCTGGTCCAG	[28]	101	AY216594.1
*Interferon gamma (IFN-γ)*	F: AAGGGCTGTGATGTGTTTCTG R: TGTACTGAGCGGCATTACTCC	[29]	68	AY795563
*Virus-inhibitory protein, endoplasmic reticulum-associated, interferon-inducible (viperin)*	F: AGCAATGGCAGCATGATCAG R: TGGTTGGTGTCCTCGTCAAAG	[30]	101	NM_001140939.1
*Myxovirus resistance (Mx)*	F: TGCAACCACAGAGGCTTTGAA A: GGCTTGGTCAGGATGCCTAAT	[31]	78	U66476.1
*Major histocompatibility complex class I* (*MHC-I*)	F: GAAGAGCACTCTGATGAGGACAG R: CACCATGACTCCACTGGGGTAG	[30]	112	EB174276
*Cluster of differentiation 8 alpha (CD8a)*	F: CGTCTACAGCTGTGCATCAATCAA R: GGCTGTGGTCATTGGTGTAGTC	[30]	118	AY693393.1
*Granzyme A (gzma)*	GGTGTTTCTAGGGGTCCACTC TGCCACAGGGACAGGTAACG	[32]	193	XM_045695185.1
*Natural killer lysin (NK-lysin)*	F: TGTTCTTATGCACCACGCAA R: CGGGTATGACGCAAAACGACTA	[33]	109	NM_0011411 10.1
*Major histocompatibility complex class II (MHC-II)*	F: CCACCTGGAGTACACACCCAG R: TTCCTCTCAGCCTCAGGCAG	[34]	116	X70165
*Cluster of differentiation 4 (CD4)*	F: GAGTACACCTGCGCTGTGGAAT R: GGTTGACCTCCTGACCTACAAAGG	[35]	121	BT056594
*Secretory immunoglobulin M (sIgM)*	F: CTACAAGAGGGAGACCGGAG R: AGGGTCACCGTATTATCACTAGTT	[30]	90	BT059185
*Membrane immunoglobulin M (mIgM)*	F: CCTACAAGAGGGAGACCGA R: GATGAAGGTGAAGGCTGTTTT	[36]	104	Y12457
*Secretory immunoglobulin T (sIgT-B)*	F: GAATGTTTGGGACACGGAAG R: TCACATATCTTGACATGAGTTACC	[37]	124	GQ907004.1
*Membrane immunoglobulin T (mIgT-B)*	F: GAATGTTTGGGACACGGAAG R: GCTCAGTCAGTGGGATGTTCT	[38]	98	GQ907004.1
*Mucin 2 (Muc-2)*	F: CGACTGCCACAAAGCCATTAGG R: GCGTGTTGCTGCGTGTCTT	[39]	53	XM_014183074.1
*Mucin 5 (Muc-5)*	F: CCGTGCTGGGAGACATTATGAAGT R: TGCTGGAGAGGGAAAGGGTAAC	[39]	81	JT819124.1
*Elongation factor-1alpha (EF1α)*	F: TGCCCCTCCAGGATGTCTAC R: CACGGCCCACAGGTACTG	[40]	57	BG933853
*SAV3-nsp1*	F: CCGGCCCTGAACCAGTT R: GTAGCCAAGTGGGAGAAAGCT Probe: FAM-5 -CTGGCCACCACTTCGA-3 -MGB	[25]	107	AY604235

## Data Availability

The data presented in this study are available on request from the corresponding author.

## Supplementary Materials

The following supporting information can be downloaded at https://www.mdpi.com/1999-4915/15/12/2450/s1, Supplementary Figure S1: Histopathological analysis of the pseudobranch tissues in Atlantic salmon following SAV3 challenge (by cohabitation) at 16 dpc showing no histological difference between (A) control fish (non-infected) and (B) SAV3-infected fish from HD group at 16 dpc.
