# Supplementary material for: In Situ Detection of Salmonid Alphavirus 3 (SAV3) in Tissues of Atlantic Salmon in a Cohabitation Challenge Model with a Special Focus on the Immune Response to the Virus in the Pseudobranch
Source: Viruses. 2023 Dec 15;15(12):2450. doi: 10.3390/v15122450 (PMC11080939; doi:10.3390/v15122450)
Supplement: Supplementary file 1 [file viruses-15-02450-s001.zip › viruses-2785901-supplementary.pdf]

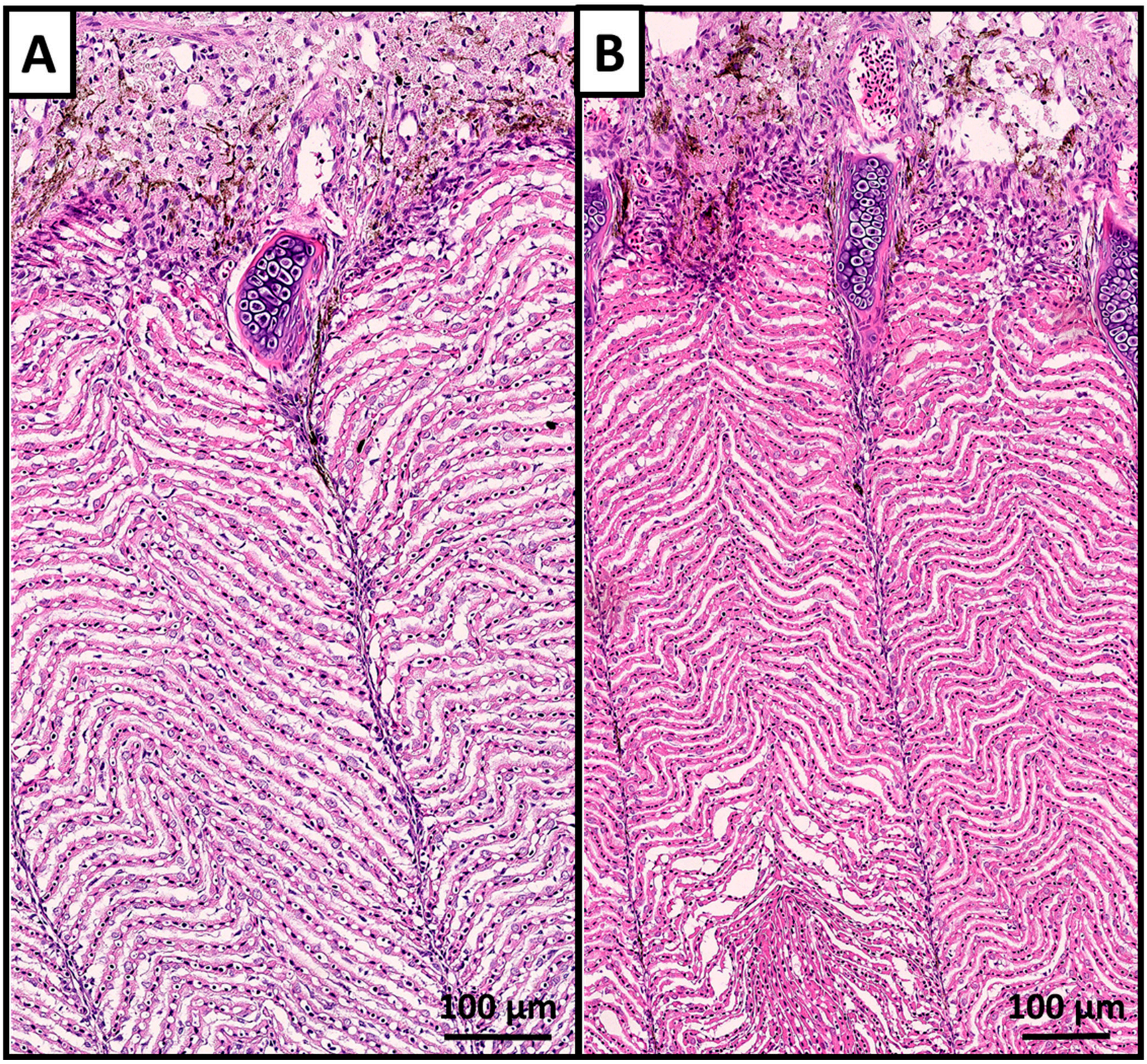

Supplementary figure 1. Histopathological analysis of the pseudobranch tissues in Atlantic salmon following SAV3 challenge (by cohabitation) at 16 dpc showing no histological difference between (A) Control fish (non infected) and (B) SAV3-infected fish from HD group at 16 dpc.
